# Changes in global and regional modularity associated with increasing working memory load

**DOI:** 10.3389/fnhum.2014.00954

**Published:** 2014-12-01

**Authors:** Matthew L. Stanley, Dale Dagenbach, Robert G. Lyday, Jonathan H. Burdette, Paul J. Laurienti

**Affiliations:** ^1^Laboratory for Complex Brain Networks, Wake Forest University School of MedicineWinston-Salem, NC, USA; ^2^Department of Psychology, Wake Forest UniversityWinston-Salem, NC, USA; ^3^Department of Radiology, Wake Forest University School of MedicineWinston-Salem, NC, USA

**Keywords:** working memory, working memory load, *n*-back, graph theory, fMRI, complex network, modularity

## Abstract

Using graph theory measures common to complex network analyses of neuroimaging data, the objective of this study was to explore the effects of increasing working memory processing load on functional brain network topology in a cohort of young adults. Measures of modularity in complex brain networks quantify how well a network is organized into densely interconnected communities. We investigated changes in both the large-scale modular organization of the functional brain network as a whole and regional changes in modular organization as demands on working memory increased from *n* = 1 to *n* = 2 on the standard *n*-back task. We further investigated the relationship between modular properties across working memory load conditions and behavioral performance. Our results showed that regional modular organization within the default mode and working memory circuits significantly changed from 1-back to 2-back task conditions. However, the regional modular organization was not associated with behavioral performance. Global measures of modular organization did not change with working memory load but were associated with individual variability in behavioral performance. These findings indicate that regional and global network properties are modulated by different aspects of working memory under increasing load conditions. These findings highlight the importance of assessing multiple features of functional brain network topology at both global and regional scales rather than focusing on a single network property.

## Introduction

The recent use of graph theory measures in complex network analyses of neuroimaging data has allowed for the identification and classification of global and regional brain network properties as well as the quantification of changes in network properties across different task conditions. In using this mathematical framework, networks are composed of differentiable elements (nodes) and the pairwise relationships between those elements (edges). In human functional brain networks, nodes represent a predefined parcellation of brain tissue, and edges represent measured functional connectivity between pairs of nodes. Approaching the study of the brain as a large-scale, interdependent network with various interacting components that produce complex behaviors offers an attractive alternative to studying the brain using univariate comparisons from activation studies or bivariate methods that exclusively identify direct functional connections between pairs of encapsulated brain regions (Telesford et al., 2011). However, the vast majority of multivariate, large-scale network analyses have only examined network properties while participants remain in a resting-state. Mapping out the relationship between network topology and cognitive tasks has been left relatively uncharted. The current work utilizes a graph theoretic complex network approach to explore a key aspect of functional brain network topology, modularity, and the relationship between module properties and behavioral performance during a working memory task in a cohort of young adults.

Working memory broadly refers to the temporary storage and active processing of information in the service of ongoing tasks (Baddeley, 1986, 1996). In addition, tasks that tap working memory often require individuals to constantly update contents in temporary storage and inhibit competing information (Jonides et al., 2003). The importance of the working memory construct is underscored by the important role it plays for optimal cognitive performance in domains as diverse as reasoning, problem solving, selective attention, general fluid intelligence, and language processing (Baddeley, 1986; Stoltzfus et al., 1996; Engle et al., 1999). A critically important feature of working memory that has received considerable attention is the quantity of information that must be held “on line” in order to effectively engage in certain tasks, commonly referred to as working memory load. Numerous neuroimaging studies have sought to localize particular brain regions activated during working memory tasks across different load conditions, consistently identifying the prefrontal cortex and parietal areas as subserving working memory functions (e.g., Braver et al., 1997; Cohen et al., 1997; Jansma et al., 2000; Veltman et al., 2003). Several other studies have measured changes in bivariate functional connections between certain regions of the working memory system as a function of working memory load, reliably identifying load-dependent increases in connectivity (Narayanan et al., 2005; Hampson et al., 2006; Axmacher et al., 2008). While these studies have produced important insights into the neural instantiation of working memory processes across load conditions, it is likely that the neural components of working memory are instantiated in a complex, interdependent manner that cannot merely be localized to particular cortical areas using univariate methodologies or sufficiently explained using bivariate methods to measure direct interactions between pairs of encapsulated brain regions.

More recent neuroimaging studies have begun to implement multivariate methodologies, such as principal component analysis, to investigate functional networks in working memory (e.g., Metzak et al., 2012; Woodward et al., 2013; Li et al., 2014), but few have taken advantage of the unique benefits conferred by a complex network paradigm utilizing graph theory measures. The present work uses graph theory measures to assess changes in modular organization as a function of working memory load. Within the realm of cognitive neuroscience, modules are taken to represent differentiable function-specific processing systems that tend to be associated with specific neural structures (Barrett and Kurzban, 2006). Using graph theory measures in complex network analyses, modules are defined as subsets of nodes within networks that are more densely interconnected among themselves than the rest of the network (Newman and Girvan, 2004; Valencia et al., 2009). The modular nature of the brain allows the system to flexibly and efficiently adapt to multiple and changing goals in the environment (Kashtan and Alon, 2005; Bassett et al., 2011), while simultaneously increasing the robustness and stability of the brain network (Solé and Valverde, 2008; Valencia et al., 2009). Properly characterizing the modular structure of the brain is crucial for understanding brain network organization during different cognitive states (Valencia et al., 2009). Effective cognitive performance on working memory tasks may necessitate the recruitment and optimal information processing of multiple modules serving different functions with weak connections between modules.

Modular properties of functional brain networks during resting-state have been identified as predictive of working memory capacity (Stevens et al., 2012), impulsivity (Davis et al., 2013), normal aging (Fair et al., 2009; Meunier et al., 2009; Onoda and Yamaguchi, 2013), and diverse neurological and psychiatric disorders (Alexander-Bloch et al., 2012; Vaessen et al., 2013; Baggio et al., 2014; Brier et al., 2014; Gamboa et al., 2014). Such resting-state analyses measure correlations in spontaneous low-frequency fluctuations in blood oxygen level dependent (BOLD) signal (Biswal et al., 1995, 1997) while participants are engaged in internally oriented mental activities in the absence of cognitively demanding, externally focused goal-directed tasks (Buckner et al., 2008; Anticevic et al., 2012). Prior resting-state analyses have identified a set of brain regions—the default mode network or DMN—that tend to be highly active, functionally interconnected, and central to global network function when individuals are not allocating attentional resources to cognitively demanding, externally focused tasks. Several graph theoretic analyses on complex networks have demonstrated that nodes within the DMN tend to be highly and consistently interconnected during resting-state (Buckner et al., 2009; Valencia et al., 2009; Cole et al., 2010; Tomasi and Volkow, 2011; Moussa et al., 2012), but topological patterns of connectivity within the DMN, as quantified by several network metrics including modularity, change from rest to task (He et al., 2009; Moussa et al., 2011; Rzucidlo et al., 2013). Importantly, however, similar patterns of activity and connectivity observed within the DMN during resting-state have also been identified during externally focused tasks that exert minimal demands on relevant cognitive resources (Andrews-Hanna et al., 2014). During external tasks that require minimal effort and attentional resources, individuals often retain the capacity to shift attentional focus toward unrelated self-generated information without substantially sacrificing cognitive performance on the external task.

While researchers have only recently begun to examine changes in modularity while participants actively engage in tasks, critical changes in the modular properties of functional brain networks have been identified across diverse tasks, including: motor learning (Bassett et al., 2011; Heitger et al., 2012), visual and auditory stimulation (Moussa et al., 2011), emotional face processing (Cao et al., 2014), odor recognition memory (Meunier et al., 2014), and decision making (Moussa et al., 2014). Particularly important are those highly connected nodes (hubs) that hold modules together providing modular structure (provincial hubs) and those network hubs that interconnect different modules allowing for the global integration of information (connector hubs; Guimerà and Nunes Amaral, 2005). Previous work examining the organization and function of hubs in brain networks has revealed the importance of hubs for promoting effective neural communication and integration of information, especially for successful cognitive performance (Bassett et al., 2009; van den Heuvel et al., 2009; Lynall et al., 2010; Cole et al., 2013; van den Heuvel and Sporns, 2013).

Few studies to date have investigated the topological properties of functional brain networks while participants engage in working memory tasks (Rzucidlo et al., 2013; Cao et al., 2014), and no studies have investigated changes in functional brain network topology as a function of working memory load. The current study fills this knowledge gap by applying graph theoretic measures of modularity and module hub properties to investigate differences in functional brain network topology as a function of working memory load. To this end, we compared modularity and module hub properties across different versions of the *n*-back task, a commonly used paradigm for the study of working memory that is particularly useful for investigating the neural basis of working memory processes (Owen et al., 2005). The task requires participants to monitor the identity of a sequence of items and to indicate whether the currently presented item is the same as the one presented *n* trials previously. Demands on working memory increase as *n* increases allowing for the exploration of the effects of increasing working memory processing load. While the 1-back version of the task primarily taps attentional resources with a simple comparison of the current item with the prior item in the sequence, the 2-back version requires many more operations. For successful performance on the 2-back task, more attentional resources must be allocated to the task and competing responses must be inhibited in order to process, temporarily store, and update constantly changing intervening items (Jonides et al., 2003; Pehlivanoglu et al., 2014).

This study investigated changes in both modularity and module hub properties of the functional brain network as a whole and regional changes in modularity and module hub properties as the demands on working memory increased from *n* = 1 to *n* = 2 on the standard *n*-back task. In investigating regional network changes, we drew primarily from previous work conducted by Rzucidlo et al. (2013), who have published data from the only other investigation of network properties in localized regions of cortex using functional brain networks while participants engage in a working memory task. Rzucidlo et al. (2013) identified a distinct shift in the location of network hubs from resting-state to the 2-back task. Specifically, they showed that during resting-state, brain network hubs were predominantly located in the DMN. During the 2-back task, the connectivity of the DMN was reduced and network hubs were predominately located within the working memory network (WMN). Because the 1-back condition is minimally demanding, participants should be capable of shifting attentional focus toward unrelated self-generated information without a substantial negative impact on performance. However, because the demands on working memory drastically increase and more attentional resources must be allocated to the external task from *n* = 1 to *n* = 2, we hypothesized that there would be clear shifts in the consistency of modular organization and the functions of module hubs within the DMN and WMN across load conditions. We also examined the relationship of behavioral working memory performance to modularity and module hub properties of the functional brain networks, both globally and within the DMN and WMN.

## Materials and methods

### Participants

A total of 14 young adults (9 females, *M*_age_ = 27.21 years, SD = 4.00, age range: 22–34 years) participated in this study. All participants were recruited via locally placed advertisements followed by a telephone screening. The Institutional Review Board of Wake Forest University School of Medicine approved this study, and all participants provided informed consent in writing. Participants were included only after fulfilling several criteria on batteries for cognition, including the Center for Epidemiological Studies Depression Scale (CES-D; Radloff, 1977) and the Modified Mini-Mental State Examination (MME/3ME; Folstein et al., 1975). Participants scoring greater than or equal to 25 of the CES-D were excluded from the study. Additionally, only right-handed participants with functional color vision (Ishihara, 1917), no history of alcoholism using the Alcohol Use Disorders Identification Test (AUDIT; Bohn et al., 1995), corrected visual acuity, and no more than moderate hearing loss were included in these analyses. These participants were originally recruited as controls in a larger study investigating the effects of aging and obesity on the brain. For that purpose, participants were recruited in three separate Body Mass Index (BMI) groups—normal weight (BMI from 18.5 to <25), overweight (BMI from 25 to <30), and obese (BMI from 30 to <40)—as part of the larger parent study. No significant systematic effects of BMI group were observed within or between conditions on any behavioral measure or network metric. As such, we combined all BMI groups for the analyses herein. All participants were paid for their participation.

### Scanning procedure

During each scanning session, fMRI data were acquired during the 1-back and 2-back tasks. All participants were provided with fMRI compatible goggles, ear plugs, and a hand-held button box custom-made to be MRI compatible and interfaced with the e-prime (Schneider et al., 2001) response box for the entirety of each session. During both 1-back and 2-back conditions, a sequence of 100 letters was presented on the screen in the goggles. The order of the letters presented in the sequence differed between participants in order to minimize a potential systematic effect of the presentation sequence. Letters appeared for 0.3 s followed by a 2.7 s blank slide during which participants were asked to respond; as such, individual trials lasted for 3 s. Each task (1-back and 2-back) lasted for a total of 5 min and 20 s. In the 1-back condition, for every letter that appeared after the first letter, participants were required to determine whether the current letter matched the previous letter in the sequence. Participants were instructed to press one button on the hand-held remote when the current letter was the same as the previous letter in the sequence. Participants were instructed to press a different button when the current letter was not the same as the previous letter. In the 2-back condition, for every letter that appeared after the second letter, participants were required to determine whether the current letter was the same as the one presented two letters back in the sequence. Participants were instructed to press one button on the hand-held remote when the current letter was the same as the one presented two letters back in the sequence. Participants were instructed to press a different button when the current letter was not the same as the one presented two letters back in the sequence. In both load conditions, participants were instructed to respond as quickly and accurately as possible with their right hands using their index and middle fingers to differentiate responses.

### Acquisition and pre-processing

For each participant, a multi-slice spoiled gradient inversion recovery (3DSPGR-IR) was used to collect high-resolution T_1_-weighted images on a 1.5T GE scanner. A GE 8 channel neurovascular headcoil was used. The protocol parameters were as follows: phase/frequency = 256/256; 156 contiguous slices, 1.0 mm thick; in-plane resolution of 0.938 mm × 0.938 mm; TE = 4.74 ms; TR = 4.68 ms; T1 = 600 ms. Blood oxygen level dependent contrast was measured using a whole-brain gradient echo echo-planar imaging (EPI) sequence with the following parameters: phase/frequency = 64/64; 159 volumes with 28 contiguous slices per volume; slice thickness = 5.0 mm; in-plane resolution of 3.75 mm × 3.75 mm; TR/TE = 2000/40 ms.

All functional data were realigned, slice-time corrected, and co-registered to a skull-stripped version of the accompanying structural data. Coregistration was checked visually for each participant. Structural data were parcellated into gray matter, white matter, and cerebrospinal maps. As part of an integrated processing procedure, all maps were warped and normalized to MNI template space (Montreal Neurological Institute^1^) using SPM8 software^2^. These acquired normalization parameters were then applied to all functional data. A band-pass filter (0.009–0.08 Hz) was applied to remove physiological noise and low-frequency drift. Six rigid-body transformation parameters generated during the realignment process and three mean signals (whole-brain, white matter, and cerebrospinal fluid) were then regressed out of the functional data. Additionally, sinus midline and sinus occipital ROIs were regressed out of the functional data. All functional data were motion corrected to eliminate scan volumes with excessive frame-wise displacement and BOLD signal change (Power et al., 2012). Values of 0.5 for frame-wise displacement and 0.5% ΔBOLD for DVARS were chosen to represent values well above the norm found in still subjects. The average number of volumes removed per subject was 1.21 for the 1-back task and 0.50 for the 2-back task.

### Generating whole-brain networks

Pre-processed functional data were masked such that only gray matter voxels were included. This was achieved by first summing the gray matter, white matter and cerebrospinal segment maps to generate a binary whole-brain mask. The mask was then intersected with gray matter areas specified by the Automated Anatomical Labeling (AAL) atlas (Tzourio-Mazoyer et al., 2002). A final step subtracts the white matter segment (thresholded at 99%) to remove subject-specific white matter edges that happened to coincide with AAL gray matter atlas.

Investigating the functional organization of the brain as a complex network allows for the mathematically rigorous quantification of differences in large-scale properties of the network as a whole and properties specific to particular regions of the network (Bullmore and Sporns, 2009; Rubinov and Sporns, 2010; Sporns, 2013). Networks are represented as graphs comprised of sets of nodes and edges. In the current study, each voxel represented a node, and functional brain networks were generated from a correlation matrix of time series data from each voxel pair using the Pearson correlation coefficient. Edge density across subjects was matched using the formula *N* = *K^S^*, with *N* equal to the number of nodes, *K* equal to the average degree, and *S* was set at 2.5. This threshold was chosen based on prior research showing that networks tend to fragment when *S* is greater than 3 (Hayasaka and Laurienti, 2010), the reproducibility of brain networks is highest at thresholds between 2 and 3 (Telesford et al., 2013), and networks with *S* = 2.5 exhibit a connection density expected based on the number of network nodes (Laurienti et al., 2011). A correlation coefficient cut-off that meets this density threshold was determined and only those correlations above the threshold were considered as functional edges in the analyses presented herein. Those edges between any two given voxels that met the threshold requirement were given a value of 1, and all other edges were given a value of 0. As such, undirected, unweighted adjacency matrices were generated for each participant representing whole-brain functional connectivity. Thresholding the network in this way ensures that comparisons are made between networks of comparable density relative to the total number of network nodes.

### Modularity

Modularity identifies the presence of subsets of nodes that are more densely interconnected among themselves than the rest of the network (Newman and Girvan, 2004; Newman, 2006). The degree to which the network can be subdivided into clearly delineated and nonoverlapping modules is quantified by the modularity *Q* statistic. The modularity value assigned to a given partition using this measure is as follows:

$$Q=\sum_{i=1}^{k}\left[\frac{e_{ij}}{M}-{\left(\frac{a_{i}}{M}\right)}^{2}\right]$$

where *e_i,j_* is a measure of within module connections in module *i*, *a_i_* is the total degree of module *i*, and *M* is equal to the degree of the entire network. Modularity algorithms are designed to maximize the value of *Q*. Because the determination of the optimal modular structure of a network is a computationally intractable problem (Brandes et al., 2008), several algorithms have been developed to balance the optimization of *Q* with run time. In the current study, the Louvain algorithm (Blondel et al., 2008) was used because it is particularly effective for efficiently quantifying modular organization in large networks. The Louvain algorithm was applied ten times for each brain network; the run that produced the partition with the highest *Q* value for the network was chosen as the best partition. Higher values of *Q* indicate a more strongly defined modular structure in the network.

**Figure 1:**
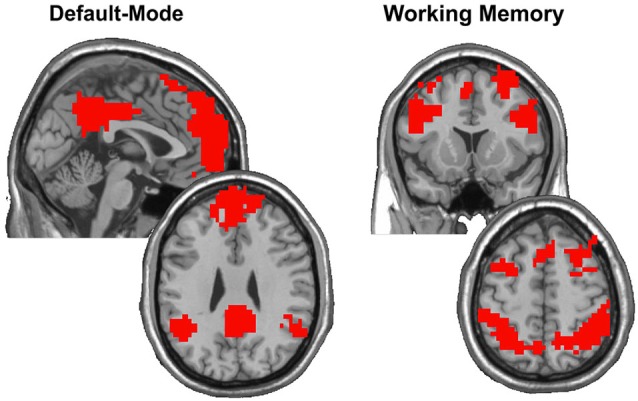
**After computing SI on the group data obtained from Rzucidlo et al. (2013) with each participant contributing five runs during resting-state and five runs during the 2-back condition, modules were identified that encompassed the DMN and WMN, respectively**. These modules were binarized to focus on the regions that exhibited consistency across all participants in each condition. The images show the regions identified as composing the DMN and WMN, respectively, which closely mirror those regions thought to subserve default mode and working memory functions across the literature.

Once modularity was computed for each individual functional brain network, the recently developed measure of scaled inclusivity (SI) was used to identify the consistency of modular organization across a specified set of participant functional brain networks (Steen et al., 2011). Scaled inclusivity is calculated by identifying the overlap of modules across multiple networks in a standardized space while penalizing for the disjunction of modules. For instance, suppose node V is part of module A in participant *i* and module B in participant *j*. Then, SI for node V is calculated as

$${\text{SI}}_{\text{V}}=\frac{\left|S_{\text{A}}\cap S_{\text{B}}\right|}{\left|S_{\text{A}}\right|}\frac{\left|S_{\text{A}}\cap S_{\text{B}}\right|}{\left|S_{\text{B}}\right|}$$

where *S*_A_ and *S*_B_ denote sets of nodes in modules A and B, and |·| denotes the cardinality of the set. Scaled inclusivity images represent the similarity in location and size of functional network modules across a set of individual participant networks (Steen et al., 2011; Moussa et al., 2012, 2014). Scaled inclusivity is calculated for each network node, and the SI value assigned to each node measures how consistently that node participates within a particular module across participants. Higher values of SI for a given network node indicate that the node is more frequently located within the same module across participants, while lower values of SI indicate that the node is more frequently located within different modules across participants. Scaled inclusivity values were calculated using DMN and WMN templates acquired from Rzucidlo et al. (2013) as referents. Figure 1 shows the location and extent of the regions included in the DMN and WMN, respectively. The resulting SI maps were compared across working memory load conditions using a modified permutation test (Simpson et al., 2013). The original form of this permutation procedure only compares the location of modules across participants using binary images. The Jaccardized Czekanowski index (Schubert, 2013; Schubert and Telcs, 2014) was incorporated to extend this statistic to include SI magnitude in addition to spatial location.

### Modular hub organization

Once the optimal modular partition was identified using the Louvain algorithm, each node’s pattern of connections relative to this partition was quantified using a method called functional cartography (Guimerà and Nunes Amaral, 2005). Particularly important are those network hubs highly interconnected with the immediate community that hold modules together and provide modular structure (provincial hubs) and those that interconnect different modules allowing for the global integration of information (connector hubs). Functional cartography has become a predominant means of identifying and classifying hubs within modules by associating two parameters to each node in the network: the participation coefficient (*pc_i_*) and within module degree (*pk_i_*). The participation coefficient is a metric that expresses the distribution of a node’s connections across all modules in a brain network. Mathematically, the extent to which a given node *i* connects to different modules is measured by the participation coefficient *pc_i_* defined as

$${pc}_{i}=1-\sum_{m=1}^{M}{\left(\frac{k_{i,m}}{k_{i}}\right)}^{2}$$

where *k_i,m_* is the number of links of node *i* to nodes in module *m*, and *K_i_* is the degree of node *i*. The closer the *pc_i_* value is to 0, the more connections that node has within a single module; the closer the *pc_i_* value is to 1, the more connections that node has to different modules in the network relative to connections within the module to which that node belongs. The *p*-value, *pk_i_* was used to accurately represent within module degree determined by 1 minus the cumulative distribution function of within module degrees (Joyce et al., 2010). In this study, those nodes with a *pk_i_* 0.05 were identified as hubs. Using the participation coefficient and within module degree measure, different classes of hubs were identified in the network. Provincial hubs, defined as those nodes with *pk_i_* ≤ 0.05 and *pc_i_* ≤ 0.3, are high degree nodes for which the majority of connections are intra-modular connections. Connector hubs, defined as those nodes with a *pk_i_* ≤ 0.05 and 0.3 <*pc_i_* ≤ 0.75, are high degree nodes for which a substantial quantity of connections are inter-modular connections. Kinless hubs, defined as those nodes with *pk_i_* ≤ 0.05 and *pc_i_* > 0.75, are high degree nodes for which almost all of their connections are with nodes in other modules. Across all participants, only 14 kinless hubs were identified at 1-back, and only 12 kinless hubs were identified at 2-back. Due to the limited quantity of kinless hubs, we did not run any additional analyses on the spatial consistency or locations of these hubs.

### Behavioral measures of working memory

Based on work in signal detection theory (Swets et al., 1961), the best available method for quantifying performance on the *n*-back task is a total score (d′) that takes into account the range for hits and false alarms by calculating the normalized proportion of correct hits minus the normalized proportion of false alarms. d′ is calculated from the hit (H) rate and false-alarm (FA) rate using the formula d′ = *Z*_H_ − *Z*_FA_, where *Z* represents a transformation of the two distributions to generate *z*-scores of the rate of hits and the rate of false alarms (Macmillan and Creelman, 1997). The better an individual maximizes hits (and thus minimizes misses) and minimizes false alarms (and thus maximizes correct rejections), the higher the individual’s d′ score. Higher scores on the d′ measure indicate better performance on the *n*-back task (for review of the d′ measure, see Haatveit et al., 2010). After excluding those responses +/− 3 SDs from the mean for each participant in each load condition, mean response times (ms) on correct trials were also evaluated.

## Results

### Behavioral results

There was a marginally significantly difference in working memory performance (d′) from 1-back to 2-back, *t*_(13)_ = 1.78, *p* = 0.099, such that participants performed somewhat worse on the 2-back task (*M* = 3.40, SD = 1.02) than the 1-back task (*M* = 3.81, SD = 0.40). Average response times were significantly longer for the 2-back task (*M* = 420.32 ms, SD = 149.26) than the 1-back task (*M* = 332.99 ms, SD = 99.75), *t*_(13)_ = 3.62, *p* = 0.003. Taken together, these results suggest that the 2-back task was more cognitively challenging than the 1-back task.

### Modularity

Whole brain individual modularity values, as defined by *Q* scores, ranged from 0.514–0.691 for the 1-back condition and from 0.562–0.691 for the 2-back condition. Thus, modular structures at both 1-back and 2-back are confirmed by these high values of *Q* obtained for each participant. Individuals’ whole brain network modularity at 1-back and 2-back were positively correlated, *r*_(12)_ = 0.690, *p* = 0.006, indicating that higher modularity at 1-back was reliably associated with higher modularity at 2-back, and vice versa. There was no significant difference in average whole brain modularity among all participants across load conditions (*p* > 0.37).

**Figure 2:**
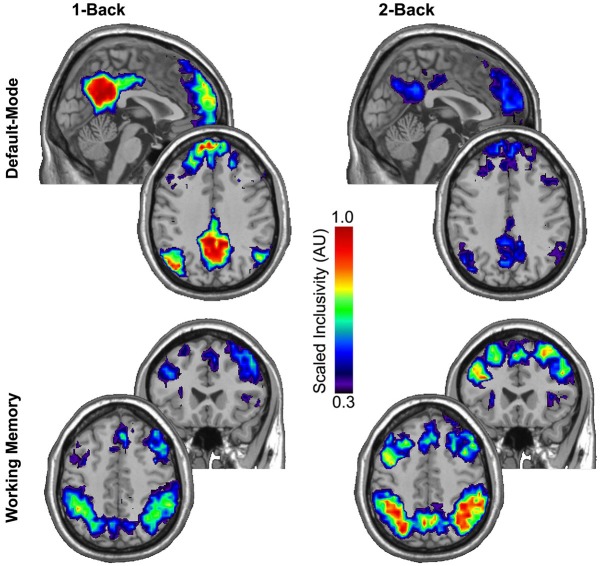
**Scaled inclusivity (SI) was used to compute the consistency of modular structure within the 1-back and 2-back conditions separately**. Higher values of SI for a given network node indicate that the node more consistently participates in the same functional module across a group of participants. The modular structure within the DMN is highly consistent across participants during the 1-back task but this consistency significantly diminishes during the more demanding 2-back task (*p* = 0.020). The consistency of modular structure across participants exhibited the opposite pattern within the WMN with significantly higher consistency in modular structure across participants during the 2-back task than the 1-back task (*p* < 0.001).

The regional consistency of modular structure across participants was measured using SI. Scaled inclusivity simultaneously identifies the degree of module overlap of nodes across participants and penalizes for any disjunction between modules. The SI maps presented in Figure 2 summarize the consistency of modular structure for the DMN and WMN across participants during the 1-back and 2-back tasks. Using the permutation framework developed by Simpson et al. (2013) in conjunction with the Jaccardized Czekanowski index (Schubert, 2013; Schubert and Telcs, 2014) to compare differences in both spatial location and magnitude of SI values, the modular structure within the DMN was identified as highly consistent across participants during the 1-back task, but that consistency in modular structure was significantly reduced during the 2-back task (*p* = 0.020). The consistency of modular structure across participants exhibited the opposite pattern within the WMN with significantly higher consistency across participants during the 2-back task compared to the 1-back task (*p* < 0.001).

### Modular hub organization

Previously established methods of functional cartography (Guimerà and Nunes Amaral, 2005; Joyce et al., 2010; Moussa et al., 2011) were implemented to evaluate differences in the quantity and spatial consistency of provincial and connector hubs as a function of working memory load. Provincial hubs, defined as those nodes with *pk_i_* ≤ 0.05 and *pc_i_* ≤ 0.3, are high degree nodes for which the majority of connections are intra-modular connections. Provincial hubs are thought to integrate processing within modules and serve to increase specificity of modular function. Connector hubs, defined as those nodes with a *pk_i_* ≤ 0.05 and 0.3 < *pc_i_* ≤ 0.75, are high degree nodes for which a substantial quantity of connections are inter-modular connections. Connector hubs are thought to integrate information between modules resulting in increased distributed information processing. This analysis revealed that there was not a significantly different quantity of provincial hubs or connector hubs in the entire brain across load conditions (both *p*’s > 0.14). The mean quantities of provincial and connector hubs during 1-back and 2-back conditions are presented in Table 1.

**Table 1:** **Mean (SD) quantities of provincial and connector hubs in the entire brain for both 1-back and 2-back conditions**.

Condition	Provincial hubs	Connector hubs
1-back	545.57 (138.07)	348.21 (132.60)
2-back	517.00 (137.87)	406.14 (117.76)

Further analyses revealed neither a significant difference in the quantity of provincial hubs within the DMN across conditions (*p* > 0.29) nor a significant difference in the quantity of provincial hubs within the WMN across conditions (*p* > 0.41). However, there was a significant increase in the quantity of connector hubs within the DMN from 1-back to 2-back, *t*_(13)_ = 5.29, *p* < 0.001. Similarly, there was a significant increase in the quantity of connector hubs within the WMN from 1-back to 2-back, *t*_(13)_ = 7.23, *p* < 0.001. The mean quantities of provincial and connector hubs within the DMN and WMN during 1-back and 2-back conditions are presented in Table 2. These data show that despite the fact that there are no significant changes in the quantity of connector hubs in the entire brain across load conditions, there is a substantial increase in the quantity of connector hubs within both the DMN and WMN as load increases. While the quantity of module hubs that serve to integrate processing within modules and improve the specificity of modular function remains relatively stable across load conditions in both the DMN and WMN circuits, there is a clear shift toward integrating information and distributing information processing between modules in the brain network serving different information processing functions as task demands increase.

**Table 2:** **Mean (SD) quantities of provincial and connector hubs within the DMN and WMN circuits for both 1-back and 2-back conditions**.

Condition	DMN provincial hubs	DMN connector hubs	WMN provincial hubs	WMN connector hubs
1-back	54.00 (32.39)	30.07 (19.86)	55.79 (26.41)	23.79 (13.98)
2-back	46.00 (22.58)	73.43 (28.14)	48.86 (24.80)	81.21 (26.80)

**Figure 3:**
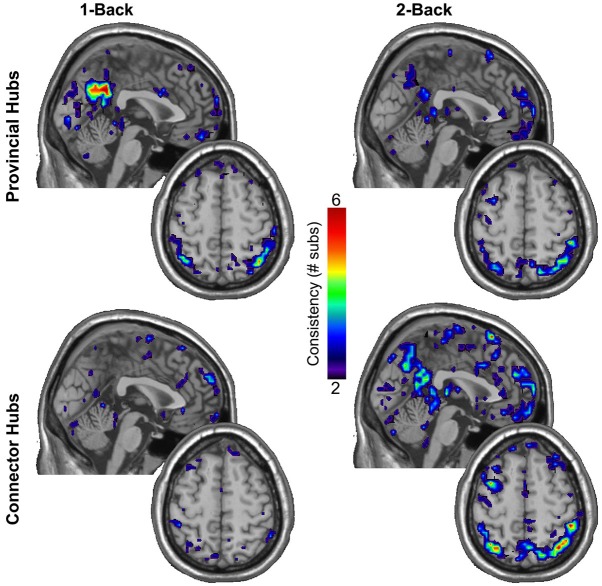
**Individual participant provincial hub (*pk_i_* ≤ 0.05 and *pc_i_* ≤ 0.3) and connector hub (*pk_i_* ≤ 0.05 and 0.3 < *pc_i_* ≤ 0.75) maps were summated within each working memory load condition separately to create an overlay image of the spatial distribution of modular hubs in standardized brain space**. Note the high consistency of provincial hubs within the ventral PC and PCC, critically important hubs of the DMN, during the minimally demanding 1-back condition, but the absence of consistency in the spatial location of provincial hubs during the 2-back condition. In addition to the sizeable increase in the total quantity of connector hubs within the WMN with increased load, there was also a significant increase in the spatial consistency of connector hubs within the WMN across participants.

In light of the substantial increase observed in the total quantity of connector hubs in both the DMN and WMN with increasing load, we further sought to explore the consistency in spatial location of provincial and connector hubs across participants within the DMN and WMN. Figure 3 shows maps of the consistency of provincial and connector hubs across participants during 1-back and 2-back conditions. Despite no significant change in the total quantity of provincial hubs in either the DMN or WMN with increasing load, there was a clear difference in the consistency in spatial location of provincial hubs across participants. During the 1-back condition, the ventral precuneus (PC) and posterior cingulate cortex (PCC) were identified as regions of the DMN with a highly consistent presence of provincial hubs across participants. However, the PC and PCC no longer displayed any consistency in the location of provincial hubs across participants during the 2-back condition. Using the permutation framework developed by Simpson et al. (2013) in conjunction with the Jaccardized Czekanowski index (Schubert, 2013; Schubert and Telcs, 2014), there was a significant decrease in the spatial consistency of provincial hubs in the DMN from 1-back to 2-back (*p* = 0.014). Overall, Figure 3 qualitatively shows that the spatial distribution of connector hubs across the brain were fairly inconsistent across participants during both 1-back and 2-back conditions, especially in comparison to the high level of consistency of provincial hubs in the PC and PCC during the 1-back condition. However, there was still a significant increase in the spatial consistency of connector hubs within the WMN across participants from 1-back to 2-back (*p* = 0.001). This change in consistency was undoubtedly aided by the fact that there were very few connector hubs located in the WMN during 1-back, whereas there was a sizeable increase in the total quantity of connector hubs across participants during 2-back. A much larger quantity of connector hubs in the WMN during 2-back (see Table 2 for details) should increase the probability of obtaining overlap in the spatial location of connector hubs. These results further suggest that the roles of certain individual nodes in the brain network are dynamic in nature and capable of functional reconfiguration in accordance with working memory load demands.

### Working memory performance and modularity

**Figure 4:**
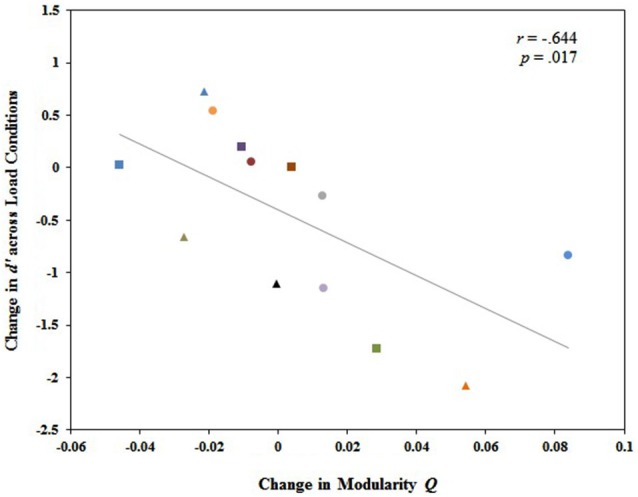
**Change in whole brain modularity from 1-back to 2-back (*Q* value at 2-back minus *Q* value at 1-back) was significantly and negatively related to the change in working memory performance from 1-back to 2-back (2-back *d’* minus 1-back *d’*)**. Each participant was identified by a unique shape and color on the graph. Participants represented by these same shapes and colors in Figure 5 correspond to the same participants in Figure 4.

We further investigated the relationship between changes in modularity across load conditions and individual differences in behavioral performance. Our analysis revealed no significant relationship between the change in modularity *Q* values from 1-back to 2-back and the change in behavioral performance (d′) from 1-back to 2-back, *r*_(12)_ = −0.421, *p* = 0.13. However, diagnostic assessments revealed a single outlier exerting substantial leverage over the slope of the least squares line. After excluding that single case from the data set, there was a significant correlation between the change in modularity *Q* values from 1-back to 2-back (2-back *Q* minus 1-back *Q*) and the change in d′ from 1-back to 2-back (2-back d′ minus 1-back d′), *r*_(12)_ = −0.644, *p* = 0.017. Those who exhibited larger magnitude increases in modularity from 1-back to 2-back also tended to exhibit larger magnitude declines in behavioral performance from 1-back to 2-back, and vice versa (Figure 4). This suggests that a global change in modularity is associated with individual variability in working memory performance. A less defined modular structure facilitating greater integration of information between modules is associated with better working memory performance.

**Figure 5:**
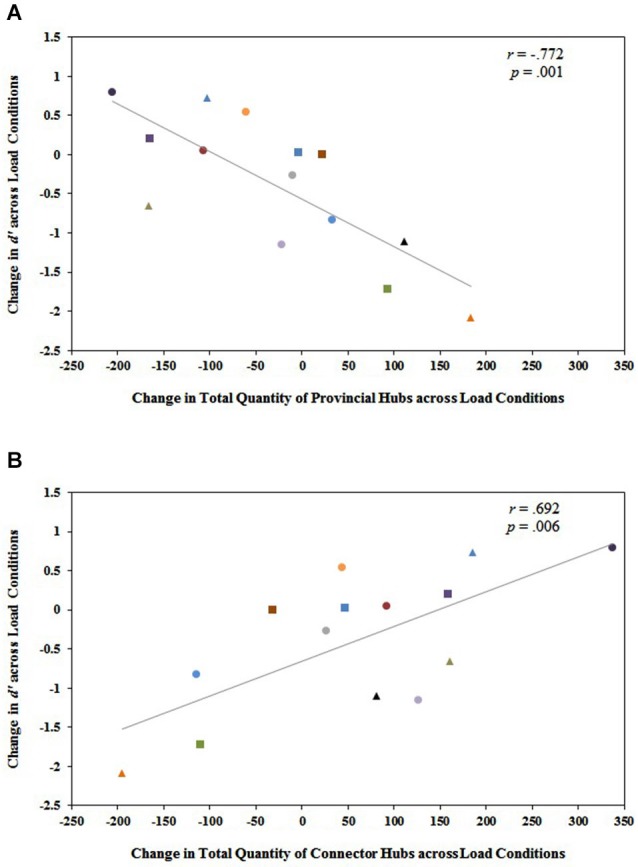
**(A)** The change in the total quantity of provincial hubs in the entire functional brain network from 1-back to 2-back (quantity at 2-back minus quantity at 1-back) is significantly and negatively related to the change in working memory performance from 1-back to 2-back (2-back d’ minus 1-back d’). **(B)** Conversely, the change in the total quantity of connector hubs in the entire functional brain network from 1-back to 2-back (quantity at 2-back minus quantity at 1-back) is significantly and positively related to the change in working memory performance from 1-back to 2-back (2-back d’ minus 1-back d’). Each participant is identified by a unique shape and color on the graph. These same shapes and colors correspond to the same participants in Figure 4. Notice that individuals who exhibited substantial decreases in modularity from 1-back to 2-back in Figure 4 tended to be the same individuals who exhibited increases in the total quantity of connector hubs from 1-back to 2-back and decreases in the total quantity of provincial hubs from 1-back to 2-back in Figure 5. These were the same individuals who actually improved their performance on the *n*-back task even though demands on working memory increased from *n* = 1 to *n* = 2. This suggests that a shift to more integrated, distributed processing is necessary for optimal working memory performance as working memory load increases.

However, no significant correlations were obtained between the change in average SI value within either the DMN or WMN across load conditions and the change in behavioral performance across load conditions (both *p*’s > 0.97).

### Working memory performance and modular hub organization

Investigating the relationship between changes in modular hub organization across load conditions and individual differences in behavioral performance, we found a significant negative correlation between the difference in the total quantity of provincial hubs in the brain network from 1-back to 2-back (quantity at 2-back minus quantity at 1-back) and the change in working memory performance from 1-back to 2-back (2-back d′ minus 1-back d′), *r*_(12)_ = −0.772, *p* = 0.001 (Figure 5). Larger magnitude declines in working memory scores from 1-back to 2-back were strongly associated with larger magnitude increases in the total quantity of provincial hubs from 1-back to 2-back, and vice versa. There was also a significant positive correlation between the difference in the total quantity of connector hubs in the brain network from 1-back to 2-back (quantity at 2-back minus quantity at 1-back) and the change in working memory performance from 1-back to 2-back (2-back d′ minus 1-back d′), *r*_(12)_ = 0.692, *p* = 0.006 (Figure 5). Larger magnitude declines in working memory scores from 1-back to 2-back were strongly associated with larger magnitude decreases in the total quantity of connector hubs from 1-back to 2-back, and vice versa.

However, no significant correlations were obtained between changes in the quantity of provincial hubs or connector hubs within the DMN across load conditions and the change in working memory performance across load conditions (both *p*’s > 0.11). Similarly, no significant correlations were obtained between changes in the quantity of provincial hubs or connector hubs within the WMN across load conditions and the change in working memory performance across load conditions (both *p*’s > 0.45). These results suggest that a more global change in the connectivity profiles of module hubs across load conditions is related to individual variability in the change in working memory performance.

## Discussion

This study utilized data-driven methods and graph theory measures to quantify changes in functional brain network topology as a function of working memory load. These analyses were made possible by recent advances in the study of complex brain networks that allow for the systematic investigation of global and regional module properties implicated in working memory. No significant changes in modularity or module hub properties were observed as a large-scale property of the brain network as a whole when working memory load on the *n*-back task increased from *n* = 1 to *n* = 2. However, significant regional changes in network topology were observed within DMN and WMN circuits across working memory load conditions. The modular organization of the DMN became significantly less consistent across participants from 1-back to 2-back, whereas the modular organization of the WMN became significantly more consistent across participants from 1-back to 2-back. Furthermore, while the quantities of provincial hubs (highly connected nodes that integrate processing within modules and serve to increase specificity of modular function) in the DMN and WMN were similar and relatively stable from 1-back to 2-back, the quantities of connector hubs (highly connected nodes that integrate information between modules resulting in increased distributed information processing) in both the DMN and WMN drastically increased from 1-back to 2-back. Despite clear shifts in regional modular consistency and module hub properties within the DMN and WMN from 1-back to 2-back and the absence of significant change in modularity and module hub properties in the entire brain network across load conditions, only the slight changes in modularity and module hub properties in the entire brain were significantly associated with individual variability in the change in behavioral working memory performance from 1-back to 2-back. In sum, while regional changes in modularity and module hub properties were highly sensitive to increases in external cognitive demands, only global changes in modularity and module hub properties were sensitive to changes in behavioral performance across load conditions.

Regarding changes in the entire functional brain network from 1-back to 2-back, a more weakly defined modular structure signifying greater integration of information between modules, an increase in connector hubs, and a decrease in provincial hubs collectively facilitate improvements in behavioral performance as working memory load increases. Taking Figures 4, 5 together, those individuals who exhibited decreases in modularity from 1-back to 2-back in the entire brain network tended to be the same individuals who exhibited increases in the total quantity of connector hubs and decreases in the total quantity of provincial hubs from 1-back to 2-back. These were the same individuals who displayed high performance on the 2-back task, and often individuals who actually improved their performance as working memory load increased. These findings suggest that the recruitment of the relevant cognitive resources associated with higher levels of working memory load requires more integrated, distributed processing between network modules for optimal working memory performance. Individual nodes in the brain network are capable of functional reorganization, and the manner in which nodes reorganize their functions has a substantial impact on individual variability in behavioral performance. The fact that only changes in whole brain network measures accounted for substantial individual variability in working memory performance complements prior working indicating that organizational properties of the entire functional network are linked to intelligence (van den Heuvel et al., 2009), learning (Bassett et al., 2011; Heitger et al., 2012), and working memory capacity (Stevens et al., 2012).

An abundance of task-relevant effects support the conclusion that DMN activity is suppressed during externally focused, demanding goal-directed behaviors (Shulman et al., 1997; McKiernan et al., 2006; Binder, 2012), while WMN activity increases in accordance with increasing task demands (Cohen et al., 1997; Rypma and D’Esposito, 1999; Kondo et al., 2004; Osaka et al., 2004). Default mode network activity suppression appears to be a mechanism through which the brain suspends certain internally oriented mental activities (e.g., mind wandering) in order to optimize externally-directed cognitive functions facilitated by other neural systems (e.g., WMN) relevant to task demands (Buckner et al., 2008; Anticevic et al., 2012; Andrews-Hanna et al., 2014). As such, DMN activity is inversely related to cognitive demand, where higher cognitive demands produced by more difficult external tasks reduce activity in DMN regions in order to accommodate the increased need for task-related processing. However, during external tasks that require minimal effort and attentional resources, individuals often retain the capacity to shift attentional focus toward unrelated self-generated information without necessarily a noticeable negative impact on behavioral performance (Stawarczyk et al., 2011; Andrews-Hanna et al., 2014).

Despite a sizeable body of work reporting changes in DMN and WMN activation with changes in task demands, little is known about changes in complex patterns of functional connectivity in the DMN and WMN in accordance with task demands. Among the few studies exploring functional connectivity within the DMN and WMN as working memory load increases, results have indicated that connectivity within both the DMN and WMN increases with working memory load, although these increases in connectivity were unrelated to behavioral performance (Newton et al., 2011; Sala-Llonch et al., 2012). Our results provide evidence for an inverse relationship between the DMN and WMN in the consistency of modular organization across participants by examining complex patterns of functional connectivity. The modular structure of the DMN was identified as highly consistent across participants during the less demanding 1-back task. Because the 1-back task requires minimal effort and attention, our results suggest that: (1) the suppression of a specific modular organization subserving internally-oriented mental functions was unnecessary in recruiting the minimal cognitive resources required in performing the task; and/or (2) participants regularly shifted attentional focus toward unrelated self-generated thoughts during the task while still performing adequately. Because the 2-back task requires many more operations making the task more demanding on relevant cognitive resources, there was a significant decrease in modular consistency across participants within the DMN and a concomitant increase in modular consistency across participants in the WMN. Thus, the specific, consistent modular organization of the DMN facilitating internally oriented mental activities receded as the external task became more difficult, while the specific, consistent modular organization of the WMN associated with relevant cognitive demands became highly engaged across participants as the task became more difficult.

The status of hubs as provincials or connectors in functional brain networks is determined by their central embedding within the network. These network hubs influence other nodes in the network via their strong participation in dynamic interactions across disparate brain regions produced by neuronal signaling. Thus, the concept and function of brain network hubs are intimately related to assessments of network communication (Hagmann et al., 2008; Estrada, 2011; van den Heuvel and Sporns, 2013). An important goal of brain network analyses is to infer patterns of communication on the basis of network topology. In the current study, functional properties of module hubs within the DMN and WMN changed in similar ways as working memory load increased. A relatively consistent quantity of provincial hubs identified within the DMN and WMN facilitated the integration of information within their respective modules during both load conditions, thereby serving a similar, critical role in specialized information processing. However, an increase in the quantity of connector hubs within both the DMN and WMN facilitated the integration of information across different modules in the network during the 2-back condition, thereby sharing information across disparate elements of the system for more global communication. Taken together, these results suggest that while DMN and WMN modules retained their respective capacities for integration of information within their respective modules facilitated by the relatively stable quantity of provincial hubs across load conditions, nodes within both DMN and WMN modules acquired a larger quantity of connections to other modules in the network as load increased, thereby facilitating the transfer of information between disparate processing systems required for increasing cognitive demands.

In examining the consistency in spatial location of provincial and connector hubs across the brain network, we identified the PC and PCC as subregions of the DMN with a highly consistent presence of provincial hubs during the minimally demanding 1-back condition. However, the consistency in the location of provincial hubs in the PC and PCC was reduced across participants during the 2-back condition. Despite no significant change in the total quantity of provincial hubs within the DMN across load conditions, individual nodes within the DMN still reorganized their functions in accordance with external task demands. That is, the particular spatial distribution of provincial hubs changed from 1-back to 2-back. In line with prior work showing a high consistency of provincial hubs within the PC and PCC of the DMN during resting-state (Moussa et al., 2011), our results suggest that those provincial hubs within the PC and PCC may play an integral role in facilitating internally oriented mental activity (e.g., daydreaming) during minimally demanding tasks. When attentional resources must be allocated to more demanding external tasks, however, nodes within the PC and PCC functionally reorganize to serve different roles in the brain network.

Early definitions of modules treated such structures as completely encapsulated information processing units (Fodor, 1983) that are neutrally instantiated in localized brain regions. It is important to emphasize again that the modules identified in the current study remain integrated with one another via a complex pattern of network connections. While viewing brain networks in terms of functional modules allows for the identification of brain regions with similar features or functions, nodes within any given module still maintain weak connections with nodes in different modules. In this sense, modularity captures an important organizational principle characterizing optimal system organization: the integration of information within subsystems allows for efficient, specialized local processing while sparse connections between subsystems reduce the proliferation of noise in the system and permit the more global integration of information for complex tasks (Bassett et al., 2011; Stevens et al., 2012).

The idea that localized brain regions are functionally specialized information processing units and make specific contributions to cognitive processes is supported by a substantial body of evidence from diverse research programs. However, localized functional specialization alone cannot fully account for most aspects of brain function (van den Heuvel and Sporns, 2013). In fact, rapidly accumulating evidence has suggested that integrative processes and dynamic, complex interactions across numerous, distributed brain regions subserve visual recognition (Behrmann and Plaut, 2013), language functions (Friederici and Gierhan, 2013), cognitive control and executive functioning (Power and Petersen, 2013), emotion-cognition interactions (Pessoa, 2012), decision making processes (Moussa et al., 2014), and social cognition (Barrett and Satpute, 2013). Similarly, our results demonstrate that the degree to which modules are interconnected by central network nodes allowing for the continuous sharing of information across distributed elements of the network is critically important for successful performance on more complex tasks. Although many different modules are engaged in more cognitively demanding working memory tasks (Jonides et al., 2003), integration of information via complex interactions between modules is critically important for improvements in working memory performance, even when working memory load increases. While univariate neuroimaging studies varying working memory load have repeatedly shown that increases in working memory load are associated with increased activation across several brain regions (Cohen et al., 1997; Rypma and D’Esposito, 1999; Kondo et al., 2004; Osaka et al., 2004), relatively little is known about the relationship between activity and complex patterns of connectivity with increases in working memory load. Whether the observed global shift from provincial to connector hubs facilitating behavioral performance is associated with greater brain activity as working memory load increases remains an open, empirical question worth investigating.

Most investigations of modularity in complex brain networks have examined network topology derived from resting-state functional connectivity both in healthy individuals (Fair et al., 2009; Meunier et al., 2009; Valencia et al., 2009; Stevens et al., 2012; Onoda and Yamaguchi, 2013; Cao et al., 2014) and among those with neurological and psychiatric disorders (Alexander-Bloch et al., 2012; Vaessen et al., 2013; Baggio et al., 2014; Brier et al., 2014; Gamboa et al., 2014). More recently, changes in modular organization and certain properties of modules in functional brain networks have been observed during diverse tasks, including: motor learning (Bassett et al., 2011; Heitger et al., 2012), olfactory recognition memory (Meunier et al., 2014), decision making (Moussa et al., 2014), and visual and auditory stimulation (Moussa et al., 2011). Results from these studies have demonstrated that certain modular properties are dynamic and changing across diverse tasks. The current study complements this prior work by demonstrating that important modular properties in functional brain networks change as a function of working memory load. Additionally, the flexible adaptation in whole brain modularity and the respective changes in provincial and connector hubs in the entire functional brain network are strongly associated with behavioral performance as load increases, a finding that underscores the value of a complex networks approach for studying task-evoked functional brain data.

Using data-driven methods each voxel in the current study represented a network node, allowing for the generation of large-scale, high resolution networks, which are unbiased and unconstrained by unnecessary *a priori* assumptions that limit the potential for making new discoveries (Stanley et al., 2013). Both large-scale properties of the network as a whole and regional changes in critical features of network topology were simultaneously investigated in the current study. Simultaneously documenting both whole brain and regional differences in network topology while relating those differences to behavioral performance on the *n*-back task allows for a particularly comprehensive investigation of neural differences in working memory processes. Complex network analyses of neuroimaging data provide a research paradigm capable of simultaneously capturing both distributive, integrated information processing and localized functional specialization within the brain (Sporns, 2013). Furthermore, we not only produced the first study detailing brain network differences as a function of working memory load, but this study also complemented a growing literature showing that functional network topology changes with task demands. Nevertheless, there are two limitations worth mentioning. First, partitioning the network into a set of modules is an NP hard problem (Brandes et al., 2008), meaning that all modularity algorithms must balance the optimization of *Q* with run time. While the modularity algorithms used in the literature are unlikely to produce the best possible network partition, the number of near-optimal partitions tends to be larger for large-scale, binary networks (Good et al., 2010), such as those networks analyzed in the current study. The use of modularity also limits each network node to a single community. It is highly probable that individual nodes can belong to multiple communities simultaneously. Future methodological development is still needed to perform the type of analyses on large-scale networks used here based on networks with overlapping communities. Second, although no significant whole-brain differences were observed in modularity, the quantity of provincial hubs, or the quantity of connector hubs as a function of working memory load, the relatively small sample size may have limited power to detect group differences. Using a larger sample size, future work should seek to investigate differences in whole brain and regional network properties as working memory load increases.

## Conflict of interest statement

The authors declare that the research was conducted in the absence of any commercial or financial relationships that could be construed as a potential conflict of interest.

## Footnotes

^1^http://www.mni.mcgill.ca/

^2^http://www.fil.ion.ucl.ac.uk/spm/
